# High prevalence and molecular identification of *Contracaecum overstreeti* (Nematoda: Anisakidae) in *Mugil thoburni* from the Galápagos Islands, Ecuador

**DOI:** 10.1016/j.ijppaw.2026.101311

**Published:** 2026-10-24

**Authors:** Manuel Calvopina, Carlos Bastidas-Caldes, Fernanda Hernández-Alomía, Camila Arcos-Suárez, Karla Flores-Zambrano, Jean Hidalgo-Jiménez, Matias Vinueza- Fernández, Nicolás Rodríguez-Calderón, Micaela Fuentes-Baquerizo

**Affiliations:** a One Health Research Group, Facultad de Medicina, Universidad De Las Américas (UDLA), Quito, 170124, Ecuador; b School of Medicine, Universidad de Especialidades Espíritu Santo (UEES), Samborondón, Ecuador; c Instituto Nacional de Biodiversidad (INABIO), Quito, Ecuador

**Keywords:** *Contracaecum*, Anisakidae, *Mugil thoburni*, Fish, Food safety, Galapagos islands, Ecuador

## Abstract

Information on anisakid nematodes infecting marine fish from the Galápagos Islands is lacking despite their potential ecological and public health importance. This study investigated the occurrence, infection parameters, and molecular identity of anisakid larvae infecting *Mugil thoburni* (blacktail mullet), a commercially important fish captured by artisanal fisheries, consumed locally, and exported to mainland Ecuador. A cross-sectional survey was conducted in October 2024 and October 2025 on 185 whole, fresh, non-eviscerated fish obtained from artisanal fisheries operating in the waters surrounding Santa Cruz Island, Galápagos Archipelago. Recovered larvae were identified morphologically and characterized by multilocus molecular analysis targeting the nuclear ribosomal ITS1–5.8S–ITS2 region, the mitochondrial cox2 gene, and the nuclear EF-1α gene, followed by Sanger sequencing and phylogenetic reconstruction. Overall, 144 of 185 fish (77.8%) were infected, yielding 436 anisakid larvae with a mean intensity of 3.03 larvae per infected fish (range 1–49) and a mean abundance of 2.36 larvae per examined fish. The year-specific prevalence was 74.1% in 2024 and 81.0% in 2025. Larvae were recovered from the visceral cavity (32.4%), muscle tissue (17.8%), or both anatomical locations (27.6%). Neither infection prevalence nor parasite intensity was significantly associated with fish size or weight. Multilocus phylogenetic analyses identified all specimens as *Contracaecum overstreeti*.

The high prevalence of infection and the detection of larvae in edible muscle tissue warrant further investigations into its zoonotic potential, including experimental infectivity studies in appropriate mammalian models. Controlled studies evaluating larval inactivation by heating, freezing, drying, acidification, and salting are also needed to establish effective fish-processing measures and inform food-safety recommendations.

## Highlights

• First report of *Contracaecum overstreeti* infecting marine fish in the Galápagos Islands.
• *Mugil thoburni* showed a high *C. overstreeti* prevalence (77.8%) and infection intensity.
• Multilocus analyses (ITS, *cox2*, EF-1α) confirmed all larvae as *C. overstreeti.*
• Anisakid larvae occurred in both the body cavity and edible muscle tissue.
• Findings support One Health surveillance to protect wildlife, fisheries, and public health.

## Graphical abstract

**Figure:**
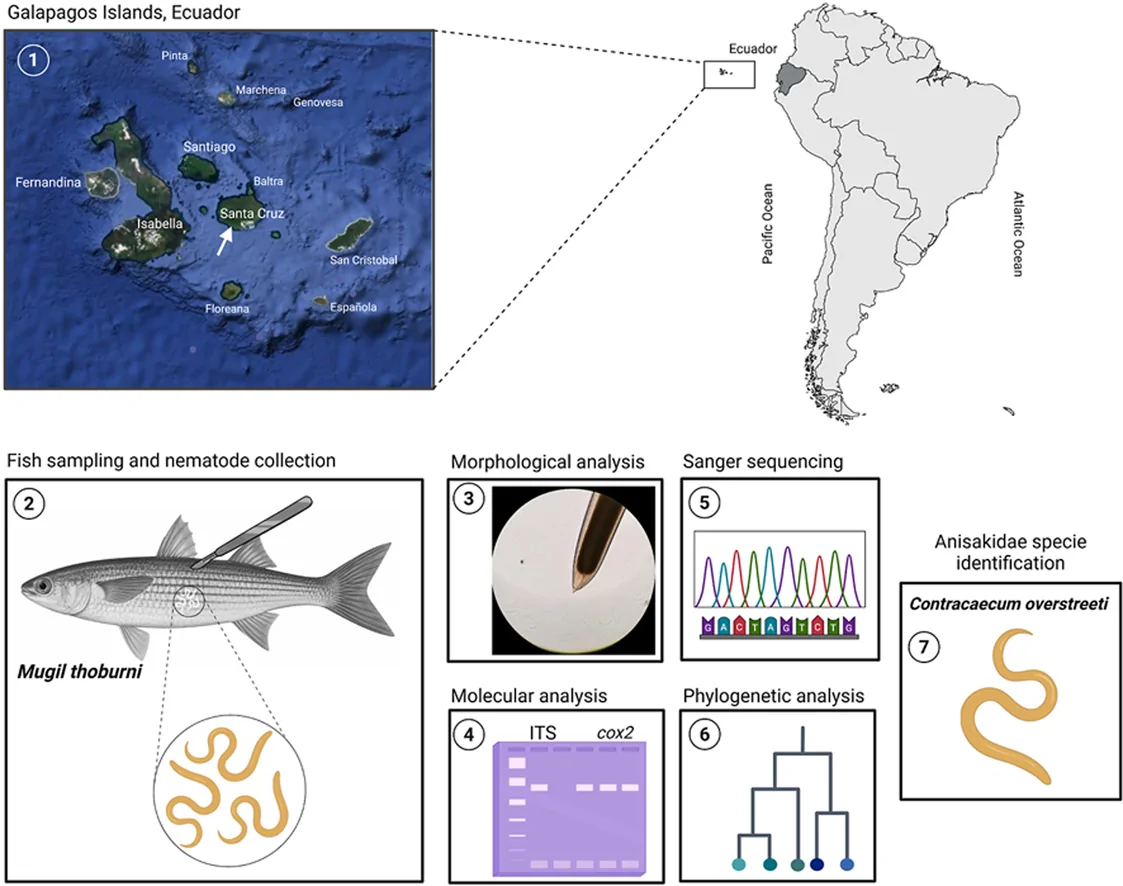

## 1 Introduction

Nematodes of the family Anisakidae are widely distributed parasites of aquatic ecosystems, with fish and cephalopods serving as intermediate or paratenic hosts for several species (Acha and Szyfres, 2003). The prevalence of anisakid larvae in fish varies considerably according to geographic region, host species, and ecological conditions, with high infection rates documented in marine fisheries worldwide (Bao et al., 2023; CDC, 2019). Certain anisakid species are recognized as zoonotic pathogens, particularly members of the genera *Anisakis* and *Phocanema* (formerly included in *Pseudoterranova*), while human infections attributed to some *Contracaecum* species have also been reported (Chai et al., 2005; Hochberg and Hamer, 2010; Shamsi and Butcher, 2011; Weitzel et al., 2015; Falla-Zuñiga et al., 2021). Recent morphological and molecular phylogenetic evidence supports the resurrection of the genus *Phocanema* for species infecting pinnipeds, including those previously assigned to the *Pseudoterranova decipiens* species complex, whereas *Pseudoterranova* is restricted to species associated with kogiid cetaceans (Bao et al., 2023). Human infections caused by certain anisakid species are of public health concern, particularly in populations consuming raw or inadequately cooked fish. Nevertheless, zoonotic potential has been established for only a limited number of anisakid species, and the capacity of *Contracaecum overstreeti* to infect humans remains undetermined.

In South America, 95 of commercially and of human consumption marine fish species had been reported infected with anisakid larvae having variable but often substantial prevalence rates (Falla-Zuñiga et al., 2021; Rave et al., 2026). Previous research conducted in central and southern coastal areas of Ecuador reported anisakid larvae in 4 of the eight commercial fish examined including bullet tuna (*Auxis rochei*), common dolphinfish (*Coryphaena hippurus*), skipjack tuna (*Katsuwonus pelamis*), and hake (*Merluccius gayi*) (Castellanos et al., 2018). A recent study in the northern Pacific Coast of Ecuador found Anisakid larvae in *Mugil cephalus* fish, (Calvopina et al., 2025). However, in Galapagos Islands, occurrence, prevalence and molecular analyses of anisakid in marine fishes are unknown (Jimenez et al., 2024). Galapagos Islands is a unique marine ecosystem characterized by high biodiversity and increasing local fish consumption.

The family Anisakidae comprises several genera of medical and veterinary importance, including *Anisakis*, *Pseudoterranova*, *Phocanema*, and *Contracaecum* (Bao et al., 2023; CDC, 2019). Investigations in South American countries including Venezuela, Colombia, Peru, and Chile have identified all three anisakid genera (Falla-Zuñiga et al., 2021). In the Pacific continental Ecuador, the first study identified *Anisakis pegreffii* and *A. physeteris* (moved to genus *Skrjabinisakis*) (Castellanos et al., 2018) and a recent study identified *C*. *overstreeti* anisakid in *Mugil cephalus* (Calvopina et al., 2025), highlighting the need for further epidemiological investigations.

Nematodes of the genus *Contracaecum* Raillet and Henry, 1912 (Anisakidae, Contracaecinae) have a worldwide distribution (Shamsi, 2019; Garbin et al., 2023). In South America, species of *Contracaecum* have been reported from both freshwater and marine ecosystems, with infections documented in commercially important fish from Argentina, Brazil, Colombia, Chile, British Guiana, Peru, Trinidad and Tobago, Uruguay and, Ecuador (Garbin et al., 2023; Calvopina et al., 2025; Villena et al., 2025; Rave et al., 2026), highlighting their broad geographic distribution and potential relevance to food safety. Several reports have shown that *Contracaecum* larvae cause human infections (Shamsi, 2019).

In Galapagos Islands, the Mugilidae fish (mullets), are popularly named as “lisa”, that are usually catched by artisanal fishery. *Mugil* spp. is one of the most ubiquitous fishes in coastal waters in most temperate, sub-tropical and tropical waters in both hemispheres. The Mugilidae family contains approximately 75 species in 17 genera. In the tropical eastern Pacific exists 8 species in three genera. The *Mugil thoburni* (Jordan and Starks, 1896) is a species found in the eastern Pacific Ocean, most common around the Galapagos Islands but does occur on the coasts of North, Central and South America as well (Crosetti and Blaber, 2016). Thoburn's mullet was the second-most numerous species in a shallow water at Isla Santa Cruz, Galápagos (Llerena-Martillo et al., 2018). The “lisa” is extensively harvested and consumed by the local population and exported to the continental land as salted dry fish. “Lisa” is prepared raw in “ceviche”, a dish prepared with raw fish marinated in lime juice, and in “fanesca” dish, prepared with dried salted fish, served during Holy Week celebrations (Calvopina et al., 2025).

Accurate identification of anisakid larvae to the species level is often difficult using morphological characters alone because of the considerable overlap in diagnostic features among genera and species (Chai et al., 2005; Shamsi and Suthar, 2016; Mattiucci and Nascetti, 2008). Therefore, molecular tools have become essential for reliable identification and phylogenetic characterization, particularly PCR and DNA sequencing of ITS rRNA and mtDNA *cox2* genes (Mattiucci et al., 2008, 2018; Zhu et al., 2002; Tunya et al., 2020; Cipriani et al., 2022; Rave et al., 2026). These approaches have significantly improved the resolution of anisakid taxonomy and have enabled the detection of cryptic species.

Given the ecological uniqueness of the Galápagos Islands and the absence of baseline data on anisakid infections in locally consumed fish, the present study aimed to determine the prevalence and to perform morphological and molecular identification of Anisakidae larvae in *Mugil thoburni*, a commonly consumed fish species captured by artisanal fisheries in this Archipielago.

## 2 Methodology

### 2.1 Study design, sampling, and study area

A cross-sectional study was conducted in October 2024 and October 2025. Fishing capture was made by artisanal fishing in the ports of Puerto Ayora of Isla Santa Cruz (0°44′40″S and 90°18′50″W) a major hub for artisanal fisheries, fish were bought from local fishers. The Galápagos Islands, located in the Pacific Ocean at the equator, about 960 km off the coast of mainland Ecuador, comprise approximately 8000 km^2^ of volcanic land, a UNESCO World Heritage Site. Administratively, the archipelago is one of the 24 provinces of the Republic of Ecuador. It consists of 13 main islands, including Santa Cruz Island, and more than a hundred smaller islets and rocks, with a resident population of around 28.583 (INEC, 2022) (Fig. 1).

**Fig. 1:**
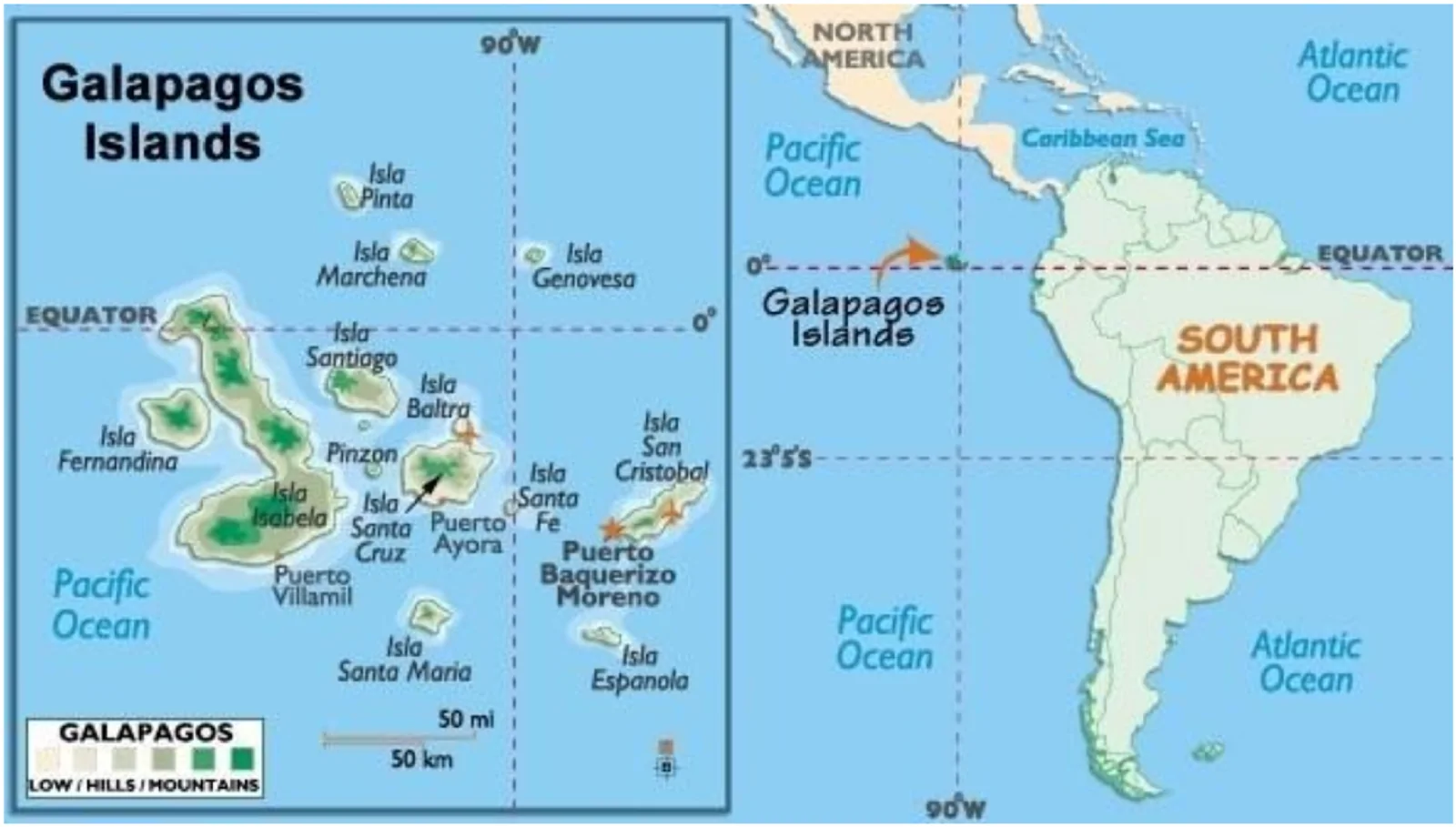
Galápagos Archipiélago, Ecuador. Black arrows indicate the Santa Cruz Island where *Mugil thoburni* specimens were captured by artisanal fishing and analyzed in this study.

### 2.2 Fish identification

Fish were randomly selected at landing sites immediately after capture to ensure freshness. Each specimen was individually labeled and transported under refrigerated conditions (4–8 °C) to the laboratory for identification and parasitological examination within 4 h. Identification of *Mugil thoburni* (Jordan & Starks in Jordan and Evermann, 1896) were identified based on external morphological characteristics following standard taxonomic keys for Mugilidae (Fischer et al., 1995; FAO, 2002). Identification was supported by the presence of a slender and elongate body, a relatively small head with a narrow interorbital region, and a terminal to slightly inferior mouth with thin lips. A key diagnostic feature was the distinctly dark to blackish caudal fin, characteristic for which is local named as “lisa rabo negro”, it differentiates from other sympatric mullets such as *Mugil cephalus* and *Mugil galapaguensis* typically characterized by a more robust body and thicker lips. Additionally, specimens exhibited a uniform silvery coloration with faint or inconspicuous longitudinal striping (Fig. 2A). Morphometric data, including total length (cm) and weight (g) were recorded for each fish prior to dissection and parasitological examination.

**Fig. 2:**
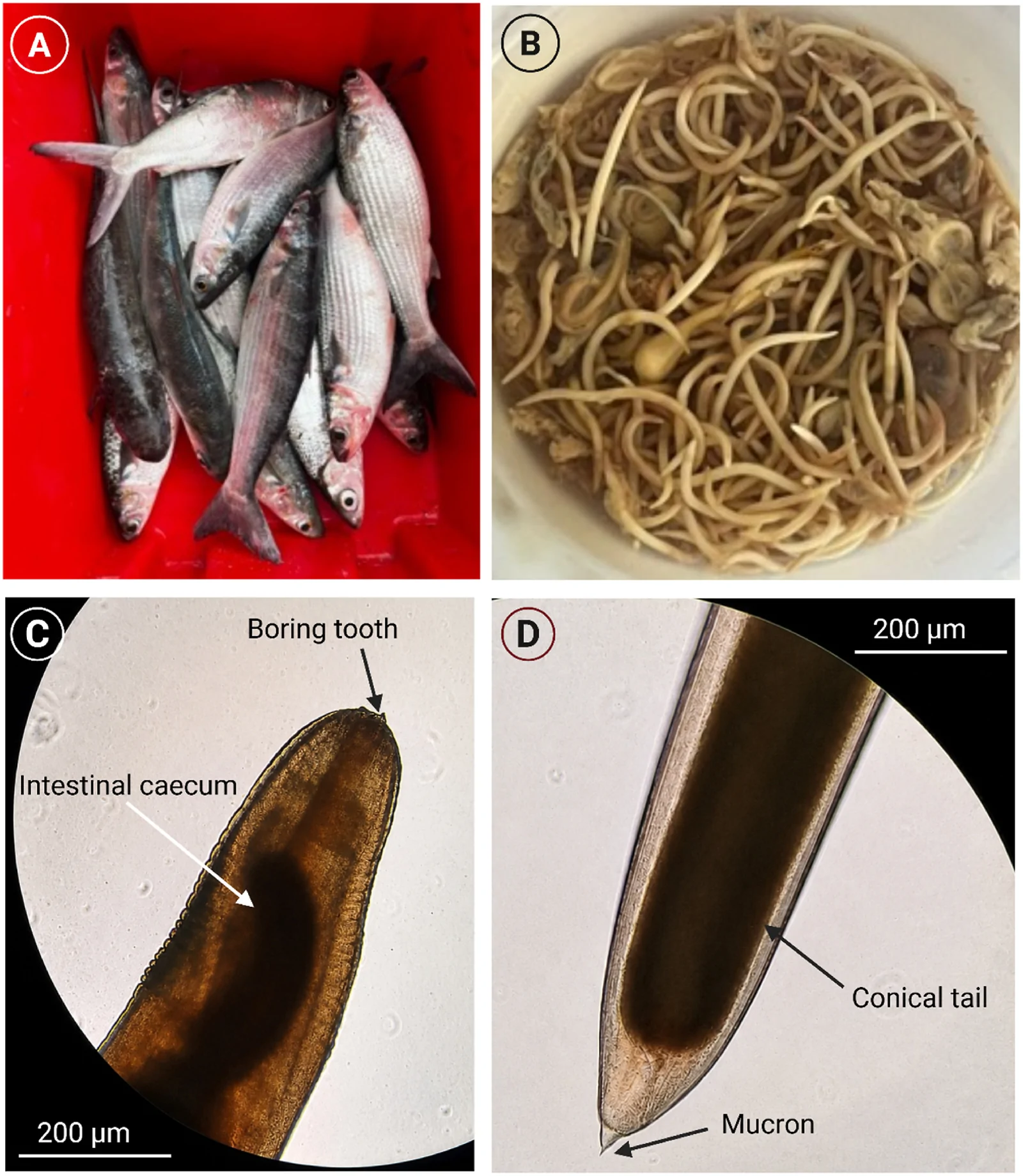
A: Fishes collected in the Galápagos Islands identified as *Mugil thoburni*. B: Larvae specimens isolated from *M*. *thoburni*. 2C and 2D: Light micrographs of anisakid larvae recovered from infected fishes. C: The cephalic extremity shows the characteristic boring tooth and three poorly developed lips, the muscular oesophagus terminates in a small, rounded glandular ventriculus and the caecum is directed anteriorly toward the cephalic end, the cuticle shown transverse striae (40x). D: Posterior extremity displaying the conical tail terminating in a pointed mucron (40x).

### 2.3 Parasitological examination

Fish were examined using a standardized manual inspection protocol. The visceral cavity was opened by a ventral incision, and internal organs were carefully inspected for the presence of nematode larvae. Subsequently, muscle tissue was examined by visual inspection to detect encysted larvae (Calvopina et al., 2025). All detected larvae were carefully removed using forceps, counted, and recorded per fish to estimate infection intensity. Larvae were classified according to their anatomical location (visceral cavity, muscle tissue or both). Recovered larvae were washed in physiological saline and preserved in 70% ethanol for further morphological and molecular analyses.

### 2.4 Morphological identification of larvae

Recovered nematode larvae were initially identified based on their morphological characteristics using the taxonomic criteria described by Ash and Orihel (1997) and standard taxonomic keys for the family Anisakidae (Shamsi, 2019; Villena et al., 2025). A representative subset of 40 larvae (20 collected in October 2024 and 20 in October 2025, recovered from the visceral cavity and/or muscle tissue) were examined by light microscopy (Zeiss Primo Star, Carl Zeiss, Germany). Morphological examination included body size and shape, cuticular ornamentation, cephalic structures (boring tooth, labial primordia, and excretory pore), digestive tract structures (oesophagus, ventriculus, anteriorly directed intestinal caecum, intestine, and posteriorly directed ventricular appendix), and morphology of the posterior extremity. These characters were used for microscopy identification of the larvae till genus level using established morphological descriptions and taxonomic criteria for Anisakidae.

### 2.5 Molecular identification

The selected 40 larvae examined morphologically were subsequently subjected to molecular analysis, including DNA extraction and PCR amplification of the ITS and *cox2* loci. Genomic DNA was extracted using the PureLink™ Genomic DNA Mini Kit (Invitrogen, Carlsbad, CA, USA) according to the manufacturer's instructions. DNA was eluted in 30 μL of AE buffer and stored at −20 °C until further processing. DNA concentration and purity were assessed using a Thermo Scientific™ NanoDrop™ spectrophotometer (Thermo Fisher Scientific, Waltham, MA, USA).

Polymerase Chain Reaction (PCR) amplification was performed targeting: 1) the ITS1–5.8S–ITS2 ribosomal region (908 bp) using primers NC5 (TAGGTGAACCTGCGGAAGGATCATT) and NC2 (TTAGTTTCTTTTCCTCCGCT) following Zhu et al. (2002); 2) a 629 bp fragment of the mitochondrial cytochrome oxidase subunit II (cox2) gene using primers 211F (TTTTCTAGTTATATAGATTGRTTTYAT) and 210R (CACCAACTCTTAAAATTATC) according to Nadler and Hudspeth (2000); and 3) a 714 bp fragment of the elongation factor 1-alpha (EF-1α) gene using *Anisakis simplex*-specific primers EF-F (TCCTCAAGCGTTGTTATCTGTT) and EF-R (AGTTTTGCCACTAGCGGTTCC), as previously described by Mattiucci et al. (2016). PCR reactions were prepared in a final volume of 15 μL containing 1X GoTaq® Green Master Mix (Promega, Madison, WI, USA), 0.2–0.45 μM of each primer, and 1 μL of template DNA. Negative controls without DNA template were included in all amplification assays. Amplification conditions followed previously described protocols (Calvopina et al., 2025).

PCR products were visualized by agarose gel electrophoresis and amplicons presenting expected fragment sizes were purified and sequenced bidirectionally using Sanger sequencing on an ABI 3500xL Genetic Analyzer (Applied Biosystems, Foster City, CA, USA) with BigDye Terminator v3.1 chemistry at the molecular biology laboratory of Universidad de Las Américas (UDLA), Quito, Ecuador. Prior to sequencing, PCR products were enzymatically purified using Exonuclease I and FastAP reagents. Raw chromatograms obtained from forward and reverse reads were assembled and manually edited in Molecular Evolutionary Genetics Analysis version X (MEGA X). Consensus sequences were subsequently compared against sequences deposited in the National Center for Biotechnology Information (NCBI) database using the Basic Local Alignment Search Tool (BLAST) for preliminary taxonomic identification.

### 2.6 Sequence alignment and refinement

Phylogenetic analyses were conducted independently for the ITS1–5.8S–ITS2 (908 bp) and mitochondrial *cox2* (629 bp) datasets. Newly generated sequences obtained in this study, together with representative anisakid reference sequences retrieved from GenBank, were aligned separately prior to phylogenetic reconstruction. Accession numbers of all reference sequences included in the analyses are provided in the phylogenetic trees and in the Supplementary Table 1. Multiple sequence alignments were generated using ClustalW and subsequently inspected manually to identify potential alignment inconsistencies and ambiguous regions. Poorly aligned terminal positions and ambiguously aligned sites were trimmed when necessary to reduce potential phylogenetic artifacts. To further improve alignment quality, curated datasets were filtered using Gblocks to remove poorly aligned regions while preserving phylogenetically informative sites. Final alignments were inspected manually before downstream analyses.

### 2.7 Maximum likelihood phylogenetic reconstruction

Maximum likelihood (ML) phylogenetic analyses were performed independently for the ITS1–5.8S–ITS2 and mitochondrial *cox2* datasets. The best-fitting nucleotide substitution models were selected according to the Bayesian Information Criterion (BIC) implemented in MEGA X prior to phylogenetic reconstruction. Phylogenetic inference for the mitochondrial *cox2* dataset was conducted under the Tamura 3-parameter model with gamma-distributed rate variation among sites (T92 + G), whereas the ITS dataset was analyzed under the Tamura–Nei model with gamma-distributed rate variation and a proportion of invariant sites (TN93 + G + I). Trees were reconstructed using the ML method with the Nearest Neighbor Interchange (NNI) heuristic search algorithm. Node support was assessed using 1000 bootstrap replicates, and values ≥ 70% were considered indicative of moderate to strong support. Sequences of *Pseudoterranova ceticola* (OP352246.1 and ON128286.1) were used as outgroup taxa to root the phylogenetic trees. Final topologies were exported in Newick format and graphically edited for figure preparation.

### 2.8 Statistical analysis

Descriptive statistics were used to estimate prevalence, mean intensity, and range of infection. Prevalence was calculated as the proportion of infected fish among the total number examined, with 95% confidence intervals (95% CI). Associations between parasite load and host variables (fish length and weight) were evaluated using appropriate statistical tests (e.g., Pearson or Spearman correlation, depending on data distribution). Statistical significance was set at p < 0.05. All analyses were performed using standard statistical software.

### 2.9 Ethical considerations

Fish examined in this study were bought from routine artisanal fisheries intended for human consumption. The study was conducted under permit issued by the Ecuadorian Ministerio del Ambiente, Agua y Transición Ecológica (MAATE-CMARG-2024-0040).

## 3 Results

### 3.1 Fish and larvae morphological identification, infection prevalence, intensity, and relationship with fish size and weight

A total of 185 fish were necropsied and examined. Eighty-five from the first collection (2024) and 100 from the second collection (2025). All were bought from the fisheries and captured by artisanal fishing in the ports of Puerto Ayora of Isla Santa Cruz (Fig. 1). Fishes were identified as *Mugil thoburni* based on morphological characteristics (Fig. 2A). Overall, 436 Anisakidae-type larvae were recovered from 144 (77,8%) of the 185 fish. All larvae were morphologically identical visualized by naked eye and under the microscope. Of the total 436 larvae, 148 (34%) were found in the visceral cavity, 56 (12.8%) were located primarily in the epaxial muscles and less frequently in the belly flaps, and 232 (53.2%) in both (Fig. 2B). From the total 436 larvae recovered during the two-year study period, 187 (42.9%) corresponded to 2024 and 249 (57.1%) to 2025. In 2024, 65 larvae were recovered exclusively from the visceral cavity, 16 from the musculature, and 106 were isolated in both anatomical locations. In 2025, the corresponding numbers were 83, 40, and 126, respectively. Overall, the proportion of muscle-only observations increased from 8.6% in 2024 to 16.1% in 2025, whereas the proportion involving both anatomical locations decreased from 56.7% to 50.6%.

All examined larvae exhibited a slender, cylindrical, cream-white body, tapering at both extremities, with a mean length of 18.26 ± 5.34 mm. The cuticle showed fine transverse annulations that were more conspicuous toward the anterior and posterior extremities. The anterior end possessed three poorly developed labial primordia and a prominent boring tooth, with the excretory pore located ventrally near the cephalic extremity. The oesophagus was narrow and terminated in a small, rounded ventriculus. A well-developed intestinal caecum extended anteriorly from the intestinal–ventricular region, whereas a distinct ventricular appendix extended posteriorly alongside the intestine (Fig. 2C). The posterior extremity was conical and exhibited a terminal mucron (Fig. 2D). However, the presence of a terminal mucron is not a universal characteristic of *Contracaecum* larvae, as caudal morphology varies among species. The combination of cephalic and digestive tract characteristics, particularly the anteriorly directed intestinal caecum and posteriorly directed ventricular appendix, supported the preliminary morphological identification of the specimens as third-stage (L3) larvae of *Contracaecum* spp. (Ash and Orihel, 1997; Mattiucci and Nascetti, 2008; Shamsi, 2014). Species-level identification as *C. overstreeti* was subsequently established through multilocus molecular analysis by using ITS, cox2, and EF-1α.

Fish total length ranged from 31.0 to 48.5 cm (mean ± SD = 39.8 ± 3.7 cm) and body weight ranged from 350 to 1100 g (mean ± SD = 690 ± 180 g). Table 1 shows its ranges and positivity for the presence of larvae and numbers (mean load). Regarding size and infection prevalence, for 2024 the prevalence varied across size categories ranging from 67.9% in the 36–40 cm group to 100% in the 45–49 cm group, despite these differences no statistically significant differences were observed among size classes (p = 0.41). For 2025, prevalence values ranged from 75.4% in the 36–40 cm group to 100% in both the 31–35 cm and 45–49 cm group, again no significant differences were detected (p = 0.62). Regarding weight and infection prevalence, for 2024 infection prevalence varied from 57.1% in the 610–900 g group to 100% in the 910–1200 g group, no statistically significant association was found. For 2025, prevalence ranged from 78.9% in the 350–600 g group to 100% in the 910–1200 g group, as in 2024, no statistically significant differences were observed among weight categories (Table 1).

**Table 1:** Show the frequency of positivity and the number of larvae (mean) found according to the different size ranges in cm and weight in g of the total 185 *Mugil thoburni*.

	n (2024)	n (2025)	Total (n)	Positive (2024)	Positive (2025)	Total Positive n (%)	p value^a^
Size (cm)							
31–35	46	3	49	36	3	39 (27.1)	
36–40	28	61	89	19	46	65 (45.1)	0.41
41–45	9	33	42	7	29	36 (25.0)	
45–49	2	3	5	2	3	5 (3.1)	
Total	85	100	185	63	81	144 (77.8)	
Weight (g)							
350–600	66	57	123	51	45	96 (67.0)	
610–900	14	37	51	8	30	38 (26.0)	0.83
910–1200	5	6	11	5	6	11 (6.6)	
Total	85	100	185	63	81	144 (77.8)	

a Fisher exact Test.

Regarding mean parasite intensity the overall values ranging from 2.58 larvae per fish in the 36–40 cm group to 4.75 in the 31–35 cm group. Higher value in 2025 (6.67), compared to 2024 (2.83) were found, no statistically significant differences in parasite intensity were detected among size categories (Kruskal–Wallis test, p = 0.57), indicating that parasite burden was relatively homogeneous across fish sizes. When stratified by weight, mean parasite intensity ranged from 2.85 larvae per fish in the 350–600 g group to 3.58 in both the 610–900 g and 910–1200 g groups. In 2024, the highest intensity was observed in the heaviest group (4.50), whereas in 2025, the 610–900 g group showed the highest value (3.80). However, these differences were not statistically significant (Kruskal–Wallis test, p = 0.83) (Table 2).

**Table 2:** Mean larval burden (intensity) according to fish size (cm) and weight (g) of the 2024, 2025 and total 185 *Mugil thoburni*.

	Mean intensity (2024)	Mean intensity (2025)	Overall mean intensity	p value^a^
Size (cm)				
31–35	2.83	6.67	4.75	0.57
36–40	2.21	2.95	2.58	
41–45	3.14	3.44	3.29	
45–49	4.00	4.00	4.00	
Total	2.03	3.41	3.12	
Weight (g)				
350–600	2.68	3.02	2.85	0.83
610–900	3.37	3.80	3.58	
910–1200	4.50	2.66	3.58	
Total	3.26	2.37	2.82	

a Kruskall Wallis.

### 3.2 Molecular characterization and phylogenetic analyses

Successful amplification of the 40 selected larvae, was obtained for all analyzed specimens using both the ITS1–5.8S–ITS2 and mitochondrial *cox2* markers. In contrast, all specimens yielded negative for the species-specific primers targeting the elongation factor 1-alpha (EF-1α) gene of *A. simplex*. A total of 19 ITS sequences (∼908 bp) and 22 *cox2* sequences (∼629 bp) of sufficient quality, were generated and deposited in GenBank under the following accession numbers. For ITS gene PZ446218–PZ446227 (year 2024) and PZ575001-PZ575009 (year 2025). For *cox*2 gene PZ472824–PZ472836 (2024) and PZ618187-PZ618195 (2025). In 2025-collect the mitochondrial *cox*2 marker revealed low levels of intraspecific variation among the nine isolates. Four haplotypes were identified. Haplotype H1 included samples A002, A003, A004, A008 and A009, whereas H2 comprised A001 and A005 samples. Isolates A007 and A010 each represented unique haplotypes, designated H3 and H4, respectively. Pairwise genetic distances among haplotypes were low, with a maximum divergence below 0.5%, indicating limited mitochondrial variability within the analyzed population. BLAST analyses of both markers consistently identified all specimens as members of the genus *Contracaecum*, with the highest sequence similarity corresponding to *Contracaecum overstreeti*. No conflicting taxonomic assignments were detected among the analyzed individuals.

Phylogenetic reconstruction based on the ITS1–5.8S–ITS2 region recovered a well-supported *Contracaecum* clade clearly separated from representatives of *Anisakis* and *Skrjabinisakis* (Fig. 3A). All ITS sequences generated in this study clustered together in a strongly supported monophyletic lineage (bootstrap = 99), forming a sister group to previously reported *C. overstreeti* sequences (MG515224 and PP737161). Other congeners, including *C. radiatum*, *C. micropapillatum*, *C. rudolphii*, and *C. gibsoni*, formed distinct and well-delimited lineages.

**Fig. 3:**
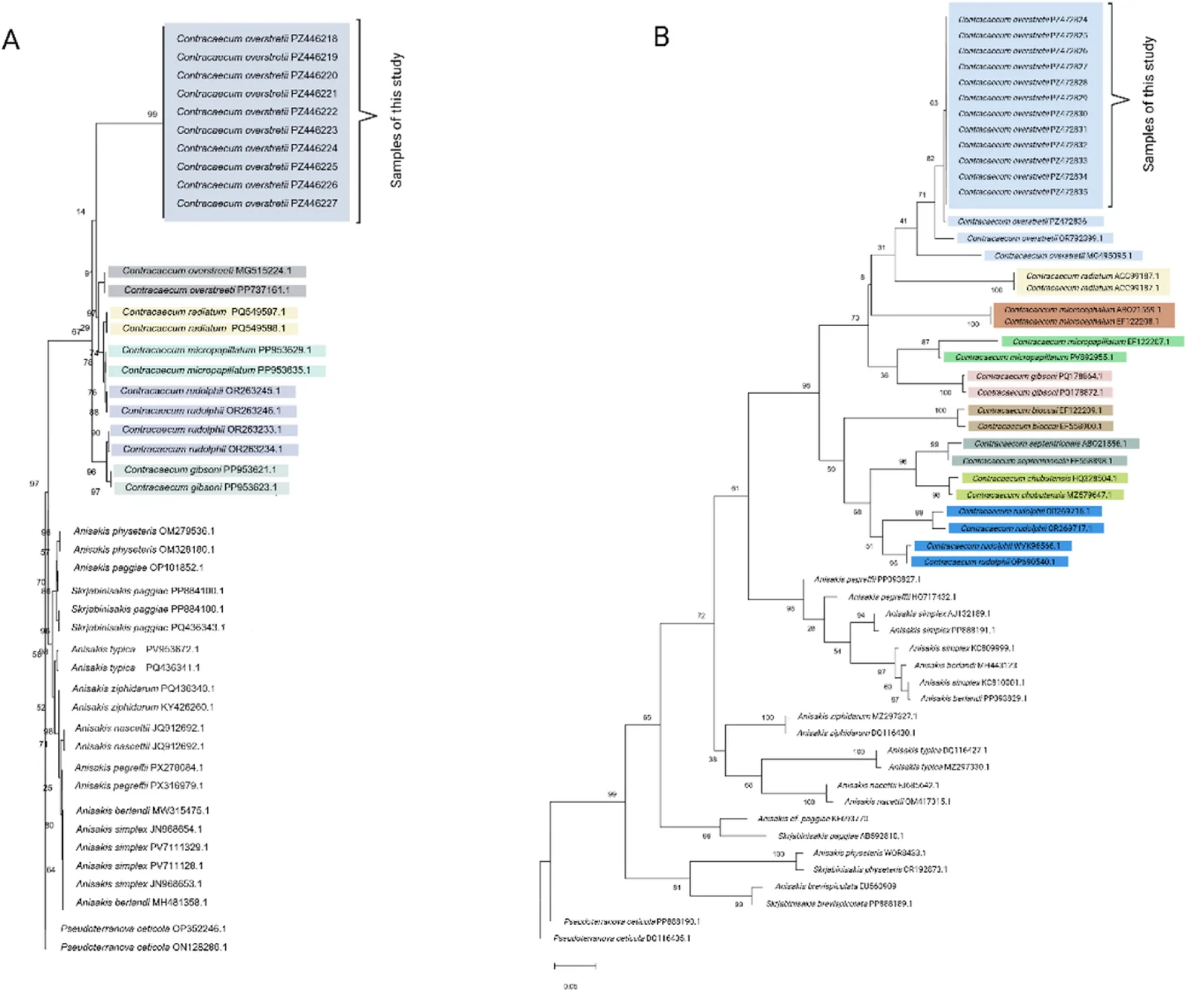
Maximum likelihood phylogenetic trees inferred from nuclear ITS1–5.8S–ITS2 (A) and mitochondrial *cox2* (B) sequences of *Contracaecum* spp. and related anisakid nematodes. Sequences generated in the present study are highlighted in sky blue color. Bootstrap support values ≥ 50%, based on 1000 replicates, are shown at the nodes. Representative sequences of *Contracaecum*, *Anisakis*, and *Skrjabinisakis* retrieved from GenBank were included for comparison. *Pseudoterranova ceticola* (OP352246.1, ON128286 for ITS and PP888190.1, DQ116435.1 for *cox2*) was used as the outgroup.

The phylogenetic tree inferred from mitochondrial *cox2* sequences produced a topology largely congruent with that obtained from the ITS dataset (Fig. 3B). Sequences generated in this study clustered within the *C. overstreeti* lineage together with reference sequences MG495095 and OR792399. Although some internal nodes exhibited moderate bootstrap support, the placement of all Galápagos specimens within the *C. overstreeti* clade was consistently recovered. Closely related species, including *C. radiatum*, *C. microcephalum*, *C. micropapillatum*, *C. gibsoni*, *C. bioccai*, *C. septentrionale*, *C. chubutensis*, and *C. rudolphii*, were resolved as independent lineages.

## 4 Discussion

This study evidence, for the first time, the occurrence, prevalence, and molecular characterization of *Contracaecum overstreeti* larvae (Nematoda: Anisakidae) in *Mugil thoburni* (blacktail mullet) from the Galápagos Islands, Ecuador. Combining parasitological analysis and molecular phylogenetics based on two independent genetic markers (nuclear ITS1–5.8S–ITS2 and mitochondrial *cox2*), larvae (L3) were identified as *C. overstreeti*.

The high prevalence of *C. overstreeti* observed during both sampling campaigns, 74.1% (63/85) in October 2024 and 81.0% (81/100) in October 2025, together with the detection of larvae in muscle tissue, highlights the potential food-safety relevance of this Anisakidae in *M. thoburni*, particularly because this fish is locally consumed prepared as “ceviche” and exported to the mainland as salted for traditional dishes such as “fanesca”. Anisakid larvae, including non-viable larvae, may contain allergens capable of eliciting allergic reactions in sensitized individuals (Audicana and Kennedy, 2008).

The prevalence estimates observed in 2024 (74.1%) and 2025 (81.0%) were high compared with several reports of anisakid infections in fish from the Americas. Recently, in the neighbouring Colombia, prevalences ranging from 13% to 100% were reported for *Contracaecum jorgei* in five freshwater fish species (Rave et al., 2026), whereas *Anisakis* larvae were reported in 33% of flathead grey mullet examined from the Colombian Pacific region (Castellanos et al., 2017). In southwestern Australia, *Contracaecum* spp. reached a prevalence of 81% in *M. cephalus* (Lymbery et al., 2002), comparable to the prevalence observed in our 2025 sampling. In Baja California, Mexico, prevalences of 30% and 14.5% were reported for two larval size classes of *C. multipapillatum* sensu lato in *M. cephalus* (Valles-Ríos et al., 2000), while a prevalence of 66.7% for *Contracaecum* sp. was recently reported in *M. cephalus* from the southeastern Pacific coast of Peru (Villena et al., 2025). Furthermore, *C. overstreeti* has recently been molecularly identified in *M. cephalus* from the Pacific coast of continental Ecuador, showing a prevalence of 90% (Calvopina et al., 2025). Collectively, these findings indicate that *Contracaecum* spp. are frequently detected in mullets and other fish hosts across different geographic regions, although direct comparisons should be interpreted cautiously because prevalence may vary according to host species, parasite species, geographic area, sampling period, sample size, and parasite detection method.

The two sampling campaigns conducted in the present study were restricted to October 2024 and October 2025. Therefore, although high prevalence was observed independently during both collections, the sampling design does not allow conclusions regarding seasonal variation, year-round persistence, or temporal stability of *C. overstreeti* infection in *M. thoburni*. Additional longitudinal sampling encompassing different seasons and multiple years would be required to characterize temporal patterns of infection in the Santa Cruz artisanal fishery.

Important to note is the absence of association between infection intensity and host biometrics. Neither fish total length nor body weight showed a statistically significant association with infection prevalence or mean larval intensity in either sampling year. However, Rave et al. (2026) reported positive correlations between host size and *Contracaecum* burden in freshwater *Mugil* spp. from Colombia, and a positive size-parasite relationship was documented in *M. cephalus* from Baja California (Valles-Ríos et al., 2000). The absence of this pattern in the present study may be attributable to some non-mutually exclusive factors: the relatively restricted body size range (subgroups) of the examined *M. thoburni* specimens (31.0–48.5 cm) or the already near-saturating prevalence across all size categories (67.9–100% depending on group).

It is worth mentioning that the distribution of larvae across the visceral cavity (34%), muscle tissue alongside the spinal column (12.8%), and both compartments simultaneously (53.2%) is a finding of particular concern from a food safety perspective, whereas most published studies on *Contracaecum* describe a predominant localization in the mesentery, coelomic cavity, and viscera (Lymbery et al., 2002; Castellanos et al., 2017, 2018; Villena et al., 2025; Rave et al., 2026). The relatively high proportion of muscle-distributed larvae observed here may relate to the advanced infection stage of *M. thoburni* at time of capture, or to host-specific factors particular to the Galápagos marine ecosystem. As in the previous study in the Pacific Coastal of Ecuador in *M. cephalus C. overstreeti* was also found in muscle tissue (Calvopina et al., 2025). Infective L3 larvae in the flesh of fish increases the risk of infection, particularly given the culinary traditions of the local population. In Ecuador, *M. thoburni* and related mullets are consumed raw in “ceviche” prepared with lime juice (Calvopina et al., 2025), a preparation that, as previously documented for *Anisakis* spp., does not inactivate anisakid larvae and therefore cannot be regarded as a food safety control measure (Falla-Zuñiga et al., 2021). Additionally, the use of salted dried fish in “fanesca” during Holy Week celebrations represents a further potential exposure route (Audicana and Kennedy, 2008).

Regarding morphological characterization, all observed larvae exhibited morphological features consistent with L3 larvae of the genus *Contracaecum*, the combination of a distinct intestinal caecum, the hallmark synapomorphy of the subfamily Contracaecinae and the pointed mucron distinguishes *Contracaecum* L3 larvae from those of *Anisakis* (which lack both a caecum directed anteriorly and a mucron in L3 larvae) and from *Pseudoterranova* (whose larvae bear a mucron but lack a well-developed intestinal caecum) (Ash and Orihel, 1997; Mattiucci and Nascetti, 2008; Shamsi, 2019). These morphological characters are consistent with previous descriptions of *Contracaecum* L3 larvae in *M. cephalus* from Ecuador (Calvopina et al., 2025), Colombia (Vergara-Flórez and Consuegra, 2021), Venezuela (Maniscalchi-Badaoui et al., 2015), and Peru (Villena et al., 2025). However, species-level discrimination within the genus *Contracaecum* is not reliably achievable by morphology alone, given the extensive overlap in metric characters among congeners at the L3 stage (Chai et al., 2005; Shamsi and Suthar, 2016).

Successful DNA amplification was achieved for all examined specimens using both the nuclear ITS1–5.8S–ITS2 and the mitochondrial *cox2* regions. BLAST analyses identified all isolates as *C*. *overstreeti*, with no conflicting assignments across individuals or markers. This dual-marker concordance substantially reinforces the species-level identification and is consistent with the approach recommended for robust anisakid identification (Zhu et al., 2002; Tunya et al., 2020). Importantly, all specimens were negative for the *Anisakis simplex* complex-specific EF-1α primers, excluding members of the genus *Anisakis* from the recovered larval population. This result contrasts with findings by Castellanos et al. (2018) who identified *Anisakis pegreffii* and *A. physeteris* (now *Skrjabinisakis physeteris*) in oceanic fishes other than Mugilidae but corroborates recent evidence for *C*. *overstreeti* in Ecuadorian Pacific waters (Calvopina et al., 2025). The molecular identification of *C. overstreeti* in *M. thoburni* from the Galápagos extends the known host range and geographic distribution of this species. The accession numbers deposited in the GeneBank will serve as an important baseline for future epidemiological and biogeographic investigations.

In the ITS1–5.8S–ITS2 tree, all Galápagos sequences clustered in a strongly supported monophyletic lineage (bootstrap = 99) as a sister group to previously deposited *C. overstreeti* sequences (MG515224 and PP737161), forming a clade clearly separated from *Anisakis* and *Skrjabinisakis* representatives. In the *cox2*-based tree, the same specimens were nested within the *C. overstreeti* clade alongside reference sequences MG495095 and OR792399, with closely related species *C. radiatum*, *C. micropapillatum*, *C. microcephalum*, *C. gibsoni*, *C. bioccai*, *C. septentrionale*, *C. chubutensis*, and *C. rudolphii* resolved as independent and well-delimited lineages. The monophyly of the *C. overstreeti* clade, consistently recovered in both datasets provides compelling phylogenetic evidence for the specific identification of all Galápagos isolates. These findings are broadly consistent with the phylogenetic framework established for the genus *Contracaecum* in South American and Pacific material (Garbin et al., 2023; Zhang et al., 2021; Calvopina et al., 2025) and affirm the taxonomic coherence of *C. overstreeti* as a genetically distinct species.

Analysis of mitochondrial *cox2* haplotypes from the 2025 collection revealed intraspecific genetic diversity with four distinct haplotypes (H1–H4), with the most abundant haplotype (H1) shared among 5 of nine individuals and haplotypes H2, H3, and H4 each represented by fewer isolates. Maximum pairwise divergence among haplotypes was below 0.5%, indicating limited mitochondrial variability within this *C. overstreeti* population. This low intraspecific divergence is consistent with observations for *Contracaecum* spp. in other geographic regions, where shallow genetic differentiation among co-occurring isolates has been reported using the same mitochondrial locus (Pekmezci and Yardimci, 2019; Zhang et al., 2021). The low haplotype diversity may reflect either a relatively recent colonization event, a population bottleneck, or ongoing gene flow mediated by the movement of migratory definitive hosts (piscivorous birds) across the eastern Pacific (Garbin et al., 2023). The presence of shared haplotypes between Galápagos isolates and previously characterized *C. overstreeti* sequences deposited from other Pacific regions would support the gene flow hypothesis; this merits investigation using larger sample sizes and additional mitochondrial or microsatellite markers in future studies.

Although the specific definitive hosts (piscivorous birds or marine mammals) of *C. overstreeti* in the Galápagos have not been investigated in the present study, the archipelago is home to a rich community of piscivorous seabirds — including the endemic Galápagos heron (*Nycticorax violaceus pauper*), lava heron (*Butorides sundevalli*), great blue heron (*Ardea herodias*), various cormorant species including the flightless cormorant (*Nannopterum harrisi*), Galápagos penguins (*Spheniscus mendiculus*), brown pelicans (*Pelecanus occidentalis*). Sea lions and seals (Pinnipeds) can serve as the primary definitive hosts for certain *Contracaecum* species (Mattiucci et al., 2008; Shamsi, 2019). Galapagos sea lion (*Zalophus wollebaeki*) is abundant lounging on beaches throughout the islands. This diversity and abundance of potential definitive hosts in Galapagos Island ecosystem is likely a primary driver of the exceptionally high prevalence observed in the present study. The fact that *M. thoburni* was identified as the second most numerous fish species in the shallow waters of Isla Santa Cruz (Llerena-Martillo et al., 2018) and is prey item for wading birds and sea lions in coastal zones further supports the ecological plausibility of intense transmission. Future studies characterizing *Contracaecum* in resident avifauna and cetaceans of the archipelago would be of considerable value for confirming the complete life cycle of *C. overstreeti* in this unique insular ecosystem.

The zoonotic potential of anisakid nematodes must be evaluated at the species level because documented human infectivity cannot be extrapolated to all members of a genus. Molecularly confirmed human infections have been reported for *Anisakis simplex* sensu stricto and *A. pegreffii* (Arizono et al., 2012; Lim et al., 2015), as well as *Phocanema decipiens* sensu stricto, previously classified as *Pseudoterranova decipiens* (Song et al., 2022; Bao et al., 2023). In contrast, evidence of human infection attributable to individual *Contracaecum* species remains limited (Shamsi et al., 2017) and requires careful evaluation of the diagnostic methods employed. Importantly, serological evidence of exposure or allergic sensitization (Salazar-Bermudez et al., 2012) does not necessarily demonstrate active infection or establish species-specific pathogenicity. Although the present study identified *C. overstreeti* larvae in edible muscle tissue of *M. thoburni*, no molecularly confirmed human infections or controlled experimental mammalian infections with this species were demonstrated in our investigation. Consequently, its ability to infect humans, invade gastrointestinal tissues, or induce clinical disease remains undetermined. The present findings should therefore be interpreted as evidence of a potential dietary exposure pathway rather than confirmation of zoonotic transmission.

The present study has some limitations that should be acknowledged. First, the cross-sectional sampling design, conducted in October of both years, does not capture potential seasonal variation in prevalence or intensity, which may fluctuate in response to changes in host abundance, definitive host migratory patterns, or ocean productivity cycles. Second, the present study was confined to a single fish species from a single Island; other Mugilidae (e.g., *M*. *galapaguensis* and *M*. *cephalus*) and additional commercially fish species captured in Galápagos waters may also require investigation. Third, the life cycle of *C. overstreeti* in the Galápagos marine ecosystem remains unknown, particularly the identity of its definitive host. Although piscivorous birds and certain marine mammals serve as definitive hosts for different *Contracaecum* species, it remains unclear whether *C. overstreeti* completes its life cycle in avian or mammalian hosts in the Galápagos Islands. Future research should include: (i) year-round longitudinal parasitological surveys to assess seasonal dynamics; (ii) parasitological screening of sympatric fish species; (iii) parasitological examination and molecular characterization of *Contracaecum* spp. recovered from resident piscivorous seabirds and marine mammals. Comparative molecular analyses of larval and adult stages would help identify the definitive hosts and clarify the transmission pathways sustaining *C. overstreeti* in the Galápagos marine food web; (iv) population genetics analyses using high-resolution markers (microsatellites, SNPs) to determine connectivity between Galápagos and continental Pacific populations; and (v) seroepidemiological and risk factors studies to estimate the actual incidence of infection in human populations where Mugilidae fish is regularly consumed raw. Forth, another limitation of this study was the use of visual inspection alone for the detection of anisakid larvae in fish musculature. Although the recovered larvae were relatively large (mean length, 18.26 ± 5.34 mm) and readily visible because of their cream-white coloration, visual examination may fail to detect larvae deeply embedded within muscle tissue. Future studies incorporating artificial pepsin digestion and UV-light examination are warranted to more accurately quantify the burden and distribution of *C. overstreeti* larvae in edible tissues of *M. thoburni*.

In conclusion, this study provides the first molecular evidence of *C*. *overstreeti* infecting *M*. *thoburni* in the Galápagos Islands, Ecuador, with high infection prevalences recorded in 2024 (74.1%) and 2025 (81.0%). The detection of larvae in edible muscle tissue identifies a potential route of dietary exposure; however, the zoonotic potential of *C. overstreeti* remains undetermined. Future investigations should prioritize experimental assessment of larval infectivity in appropriate mammalian models, where scientifically and ethically justified, and controlled evaluation of larval inactivation through heating, freezing, drying, salting, and acidification. These studies, together with species-specific epidemiological surveillance and molecular characterization of parasites from potential definitive hosts, are essential for clarifying the biological significance of *C. overstreeti* and developing evidence-based food-safety recommendations for the Galápagos Islands. An additional research priority is the experimental evaluation of processing methods capable of inactivating *C. overstreeti* larvae in edible fish tissues. Studies involving other anisakid species have demonstrated that larval survival depends on processing conditions, including temperature, exposure duration, and characteristics of the fish product (Biary et al., 2025).

From the public health perspective, these findings underline the need to strengthen preventive measures and food-safety surveillance for anisakid parasites in the Galápagos Archipelago. Public health measures should include adequate cooking or freezing of fish intended for raw or undercooked consumption, prompt evisceration and appropriate handling of freshly caught fish to minimize post-mortem migration of larvae from the viscera into edible musculature, and systematic inspection of fish destined for human consumption. Educational interventions targeting artisanal fishers, fish processors, food handlers, restaurants, and consumers should emphasize the potential risks associated with consuming raw or inadequately prepared fish and appropriate methods for preventing exposure to viable anisakid larvae. Surveillance of commercially important fish species should be complemented with epidemiological and serological studies in humans to determine the extent of exposure and assess the potential public health relevance of anisakid infection in the Galápagos Islands.

## CRediT authorship contribution statement

**Manuel Calvopina:** Writing – review & editing, Writing – original draft, Supervision, Project administration, Methodology, Investigation, Funding acquisition, Conceptualization. **Carlos Bastidas-Caldes:** Writing – review & editing, Methodology, Data curation. **Fernanda Hernández-Alomía:** Writing – review & editing, Data curation. **Camila Arcos-Suárez:** Writing – review & editing, Methodology, Data curation. **Karla Flores-Zambrano:** Writing – review & editing, Data curation. **Jean Hidalgo-Jiménez:** Writing – review & editing, Data curation. **Matias Vinueza- Fernández:** Writing – review & editing, Data curation. **Nicolás Rodríguez-Calderón:** Writing – review & editing, Data curation. **Micaela Fuentes-Baquerizo:** Writing – review & editing, Data curation.

## Ethical statement

The study was conducted under permit issued by the Ecuadorian Ministry of Environment, Water and Ecological Transition (Ministerio del Ambiente, Agua y Transición Ecológica, MAATE-CMARG-2024-0040). Ethical review and approval were not required because the investigations were performed on a dead commercial fish. No live animals were involved.

## Availability of data and materials

The sequences generated in the present study were submitted to the GenBank database under the accession numbers: ITS (PZ446218–PZ446227 and PZ575001–PZ575009); cox2 (PZ472824–PZ472836 and PZ618187–PZ618195).

## Funding

This study was supported by the grant of Universidad de las Americas (UDLA) Quito (Grant No.490.B.XIV.24 to MC).

## Conflict of interest

The authors declare no conflict of interest.
